# Trees with anisohydric behavior as main drivers of nocturnal evapotranspiration in a tropical mountain rainforest

**DOI:** 10.1371/journal.pone.0282397

**Published:** 2023-03-31

**Authors:** Volker Raffelsbauer, Franz Pucha-Cofrep, Simone Strobl, Johannes Knüsting, Michael Schorsch, Katja Trachte, Renate Scheibe, Achim Bräuning, David Windhorst, Jörg Bendix, Brenner Silva, Erwin Beck

**Affiliations:** 1 Department of Plant Physiology, University of Bayreuth, Bayreuth, Germany; 2 Chair of Atmospheric Processes, Brandenburg University of Technology (BTU) Cottbus-Senftenberg, Cottbus, Germany; 3 Grupo de Investigación Hidrología y Climatología, Universidad Técnica Particular de Loja, Loja, Ecuador; 4 Department of Plant Physiology, University of Osnabrueck, Osnabrueck, Germany; 5 Institute of Geography, Friedrich-Alexander-University Erlangen-Nuremberg, Erlangen, Germany; 6 Institute for Landscape Ecology and Resources Management (ILR), Justus Liebig University Giessen, Giessen, Germany; 7 Institute of Geography, Philipps University Marburg, Marburg, Germany; 8 Alfred-Wegener-Institute, Bremerhaven, Germany; Ningbo University, CHINA

## Abstract

This study addresses transpiration in a tropical evergreen mountain forest in the Ecuadorian Andes from the leaf to the stand level, with emphasis on nocturnal plant-water relations. The stand level: Evapotranspiration (ET) measured over 12 months with the Eddy-Covariance (ECov) technique proved as the major share (79%) of water received from precipitation. Irrespective of the humid climate, the vegetation transpired day and night. On average, 15.3% of the total daily ET were due to nocturnal transpiration. Short spells of drought increased daily ET, mainly by enhanced nighttime transpiration. Following leaf transpiration rather than air temperature and atmospheric water vapor deficit, ET showed its maximum already in the morning hours. The tree level: Due to the humid climate, the total water consumption of trees was generally low. Nevertheless, xylem sap flux measurements separated the investigated tree species into a group showing relatively high and another one with low sap flux rates. The leaf level: Transpiration rates of *Tapirira guianensis*, a member of the high-flux-rate group, were more than twice those of *Ocotea aciphylla*, a representative of the group showing low sap flux rates. Representatives of the *Tapirira* group operated at a relatively high leaf water potential but with a considerable diurnal amplitude, while the leaves of the *Ocotea* group showed low water potential and small diurnal fluctuations. Overall, the *Tapirira* group performed anisohydrically and the *Ocotea* group isohydrically. Grouping of the tree species by their water relations complied with the extents of the diurnal stem circumference fluctuations. Nighttime transpiration and hydrological type: In contrast to the isohydrically performing trees of the *Ocotea* group, the anisohydric trees showed considerable water vapour pressure deficit (VPD)-dependent nocturnal transpiration. Therefore, we conclude that nighttime ET at the forest level is mainly sourced by the tree species with anisohydric performance.

## Introduction

Nocturnal transpiration of trees and other plant life forms in various ecosystems has become an important research subject. It can account for a substantial amount of 10–25% of total daily water loss, and therefore needs to be considered in the hydrological balance of forest vegetation, especially in the tropics [1–6]. Previous studies have presented evidence for a multitude of conducive functions of the intuitively adverse coincidence of water and carbon loss during the night [7, 8]. Nighttime transpiration (NTT) can stimulate nocturnal xylem sap flux (SFn), thus helping to refill the tree’s stem water capacitance [9, 10] and to remove possible embolisms that had occurred during strong daytime transpiration [11–15]. SFn can fuel leaf cell expansion [7], transport inorganic nutrients to shoot tissues [8, 16, 17], and carry oxygen [18] for maintenance of respiration and growth during the night. Finally, opening of the stomata during the night prevents high internal CO_2_ concentrations, which might inhibit leaf metabolic activity and growth [19].

NTT corresponds to stomatal conductance, exceeding cuticular transpiration several-fold [1]. Stomatal action is endogenously controlled in a circadian mode, but in addition, responds to the water supply from the soil [3, 8, 9, 20]. Leaf age also appears to play an important role: Young leaves transpire more than adult leaves [3]. Nocturnal sap flux is driven by the water-potential gradient between the roots and the water-storing tissues that supplemented transpiration during daytime. Therefore, SFn in addition responds to the amount of water lost during the preceding light period. Besides transpiration and refilling of the trees’ water reservoirs, water pumped by root pressure may also contribute to the sap flux in the xylem [21]. In summary, the contributions of the individual endogenous and exogenous drivers that supply and regulate NTT and SFn may vary from day to day, from season to season [22–26], and from species to species [2, 27], thus hampering generalization. In a study with wine grape varieties, NTT has been brought into context with the hydricity concept [28], where anisohydricity corresponded with a statistically significant higher nocturnal transpiration and leaf conductance.

Nighttime stomatal conductance (g_n_) and the rates of SFn as well as the nocturnal share of the trees’ total daily water consumption are higher in tropical trees than in plants of other climate zones and vegetation types [3, 6]. However, most of the data from tropical trees originate from regions with a pronounced dry season, and only a few studies compared NTT during dry and wet seasons. A lower VPD during the wet season resulted in lower shares of NTT of the total daily transpiration [29]. Generally, responses of g_n_ to drought are highly variable, depending on the environmental conditions and the duration and severity of the stress. In *Eucalyptus* saplings, an increase in VPD increased of g_n_, while drying soil decreased g_n_ [30]. Given the mentioned high shares of NTT of tropical trees, the question arises of how NTT affects the dynamics of the above-canopy atmospheric moisture budget.

Endeavors to upscale NTT from the tree to the stand level have been undertaken [31] using lysimeters [32, 33], the ECov technique (e.g., [34, 35]), or modeling approaches [36–38]. Averaging ECov measurements from 99 FLUXNET-2015 sites distributed globally over various vegetation types revealed an average nocturnal share of 6.3% of total ET, while the mean of nocturnal ET determined by 26 different climate models was 7.9% [38].

So far, ECov measurements have not been conducted in the evergreen mountain forests of the tropical Andes. We are aware of one study that investigated daytime water relations of several evergreen tree species of that region at the tree and leaf level, from which ET for the stand level was extrapolated [39, 40]. A first attempt to directly measure ET in the Andean tropical mountain rainforest was undertaken in 2015 by combining area-average measurements with a laser scintillometer above the forest canopy with satellite data at a high spatial resolution [41]. ET maps were established for one day of a very humid (June) and one day of a less humid (November) month. Comparing atmospheric moisture above individual tree crowns revealed considerable differences, suggesting the co-occurrence of trees with different transpiration intensities. Due to the use of satellite data, ET could be determined only for the daylight period. However, for a realistic picture of stand ET, the contribution of NTT to the ET budget is required, which might be rather high in a mountain forest [29], especially during less humid periods.

In this study, we examined the hypotheses that i) tree species with iso- and anisohydric-like hydrology co-occur in the Ecuadorian tropical mountain forest, and ii) NTT plays a significant role for the total ET budget of the forest. To characterize the water budget of the studied trees, we considered different ecophysiological levels: gas exchange of the leaves, water consumption of the trees, and ET of the study area, with a special emphasis on the partitioning between day- and nighttime.

## Location, material and methods

### Study area

**Fig 1:**
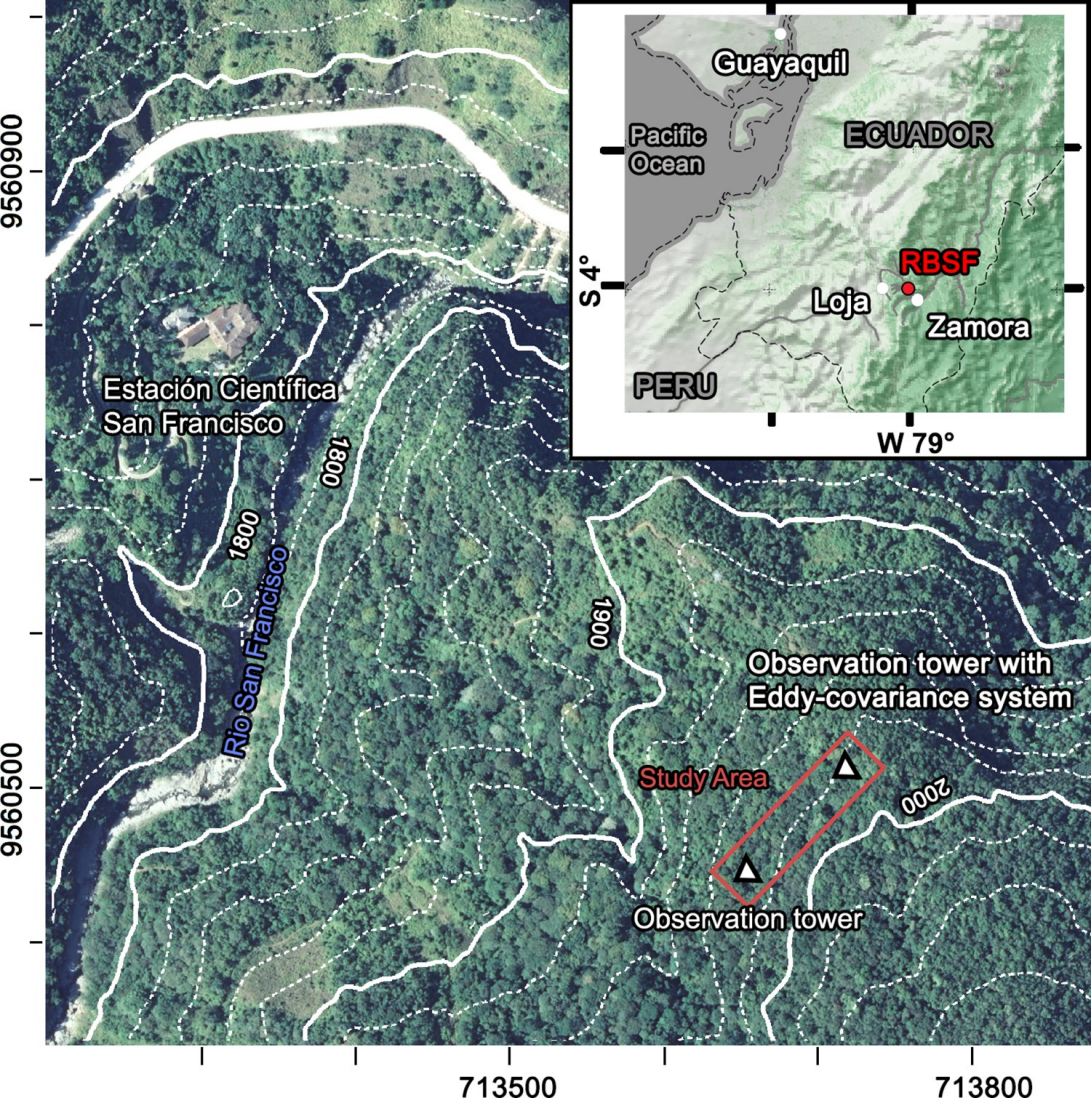
Location of the study region in southern Ecuador (top right) and of the study area in the valley of the San Francisco river (Reserva Biológica San Francisco, RBSF) with the research station Estación Cientifica San Francisco (ECSF) and the position of the core research area between two canopy towers. The Ministerio de Agricultura, Ganadería, Acuacultura y Pesca—Ecuador has kindly provided the SIGTIERRAS (Sistema Nacional de Información y Gestión de Tierras Rurales e Infraestructura Tecnológica) orthophoto, which is the basis of the figure.

The study site is located in the tropical mountain rainforest of the Reserva Biológica San Francisco (RBSF, 3°58‘26“S, 79°4‘32“W, 1970 m a.s.l.) on a north-facing slope in the Cordillera Real in the Andes of South Ecuador (Fig 1). The forest on that slope belongs to the second hottest biodiversity hotspot worldwide [42–44], with up to more than 250 woody species per hectare [45]. It is classified as an evergreen lower montane forest [46] with tree heights up to 18 m. The forest is dominated by species of the Lauraceae and Melastomataceae. The soil in this part of the forest has been characterized as humic cambisol which is covered by a raw humus layer of up to 35 cm thickness [47].

**Fig 2:**
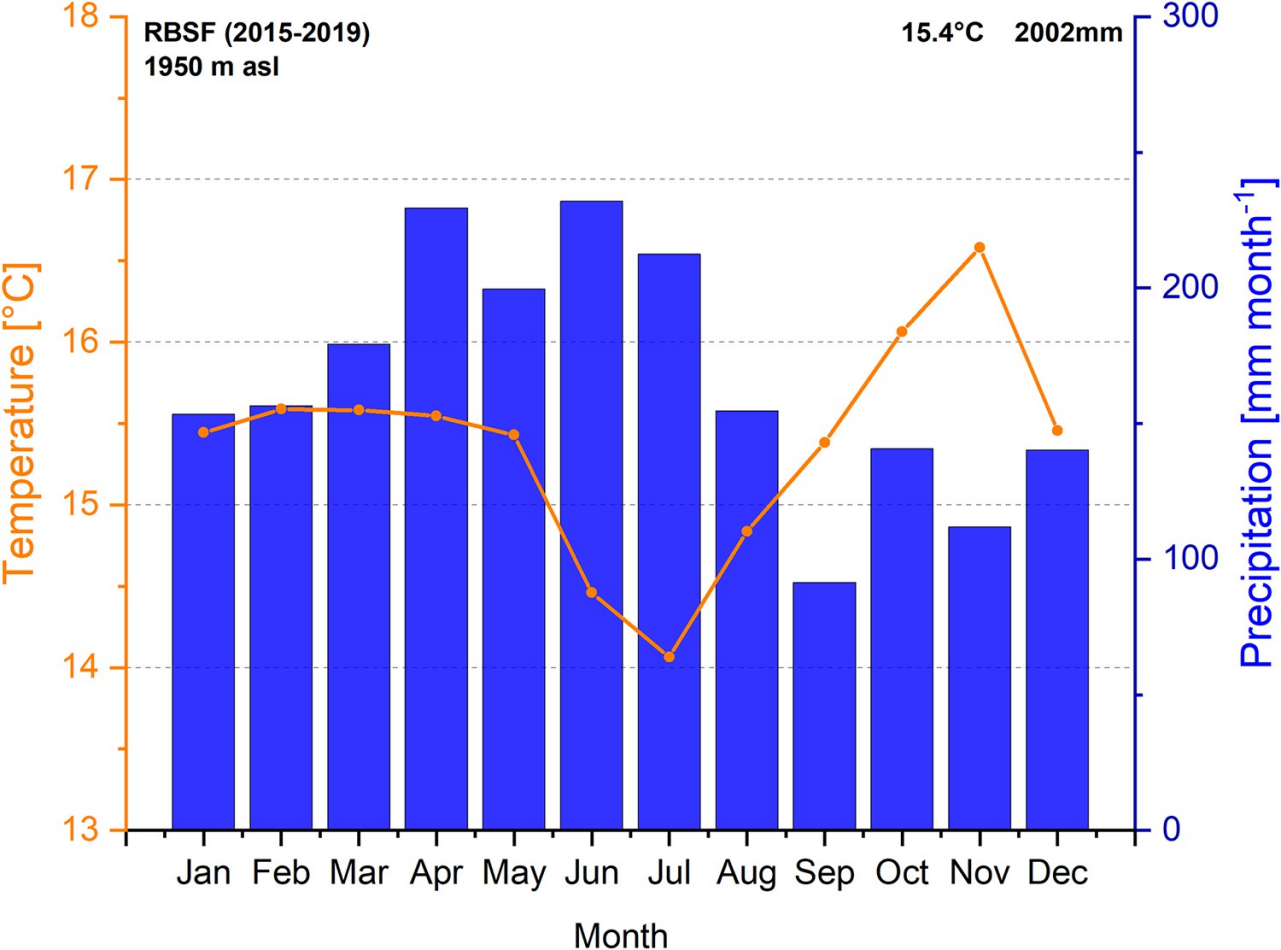
Climate diagram of the Reserva Biológica San Francisco (RBSF) during the study period (2015–2019, 1950 m a.s.l.).

Mean annual temperature and precipitation during the study period 2015–2019 were 15.4°C and 2002 mm, respectively. The climate is per-humid (Fig 2) and rain-free months are extremely rare exceptions. Precipitation is especially high from April to July, whereas less rain is recorded mostly from September to December. Due to the frequent rainfalls, the soil is permanently wet, but the topsoil may transiently desiccate, losing up to 25% of its water content.

### Research plot and tree species

The research plot consisted of a stretch of 100 m in length and up to 25 m in width, which is fully overlaid by the footprint of the ECov measurements. The footprint has a radius of around 200 m with wind speeds ranging between 3–5 m/s, within which 90% of the cumulative carbon and water signal occurs. Within the plot, two steel towers of 30 and 36 m in height were mounted, each with three platforms at 11, 14, and 21 m, respectively. Towers were used for climate and ECov measurements above the canopy and gas exchange measurements of leaves. A plain description of the trees is presented in Table 1. In addition to the measurements in the crowns, the stems of these and additional trees were equipped with sap flux sensors (see below).

**Table 1:** Characterization of the investigated trees: Height, diameter at breast height (DBH), projected crown area, specific leaf area (SLA), and leaf exposition. SLA data from personal communication by Jürgen Homeier (Dept. Resource Management, University of Applied Sciences and Arts (HAWK) Göttingen, Germany).

Tree species	DBH [cm]	Height [m]	Projected crown area [m^2^]	SLA [cm^2^g^-1^]	Leaf exposition
*Tapirira guianensis* (Anacardiaceae)	28.3	16	17.7	55.6	Sun crown
*Ocotea aciphylla* (Lauraceae)	21.0	15	11.8	47.2 57.4	Sun crown Shade crown
*Beilschmiedia tovarensis* (Lauraceae)	27.9	16	32.9	80.5	Sun crown
*Vismia canavillesiana* (Hypericaceae)	22.2	17	18.4	50.0 70.0	Sun crown Shade crown

### Eddy covariance

ET was measured with the ECov technique [48, 49] installed on the eastern of the two towers in a height level of ~25 m, which represents approximately 1.5 times the mean canopy height. The ECov system consists of a 3D sonic anemometer and an open-path CO_2_/H_2_O gas analyzer (IRGASON, Campbell Scientific, USA). The tower is also equipped with additional meteorological sensors above the canopy to measure net radiation (NR01, Hukseflux, The Netherlands), relative humidity, and air temperature (TA, Vaisala humidity and temperature probe HMP 155), as well as to derive the water vapor pressure deficit (VPD). The flux data were processed following the Fluxnet standard procedure. For subsequent analysis records with precipitation were discarded at the thirty-minute interval, as rain blurred the measurements.

### Soil water measurements

Soil water content (SWC) in m^3^/m^3^ was measured continuously in the mineral soil (below the organic layer) in intervals of 5 min between the two towers in four depths at 5 cm, 15 cm, 30 cm, and 60 cm using a Hydra Probe II (Stevens Water Monitoring Systems, Inc., Portland, USA).

### Leaf transpiration

Leaf gas exchange and conductance for water vapor of mature intact leaves in the upper crown were measured using a portable infrared gas analyzer (IRGA) photosynthesis system (LI6400 XT, LI-COR Biosciences GmbH, Lincoln, USA) equipped with a 6 cm^2^ clear bottom chamber allowing measurements at largely ambient conditions of light intensity, air temperature, relative humidity, and CO_2_ concentration (380–400 ppm). Flow rates were set to 500 μmol s^-1^. Data were recorded every five minutes starting from around sunset (18:00) for the following 24 or up to 48 hours, during which the leaves remained in the chamber. Measurements were taken on five more days (six replicates) between September 20, 2014, and December 3, 2015, with different leaves of each investigated tree. These trees were also equipped with sap flux sensors. The daily changing weather conditions (Figs 5, 7, and 8) prohibited an averaging of the six daily courses of leaf transpiration. The examples presented in Figs 7 and 8 were selected by the best match of the chamber conditions with the microclimate recorded by an external climate station (Campbell Sci. Inc., USA). The sensitivity (m) of nocturnal leaf conductance to VPD was determined according to Oren et al. (1999) [50], using the equation g_s_ = g_sref_—m x lnVPD / VPD_ref_). VPD_ref_ = 1 kPa was contained in the data set of *Tapirira* (g_sref_ represents leaf conductance at VPD = 1 kPa). In the data set of *Ocotea*, nocturnal VPD of 1 kPa was not reached and m was calculated with g_sref_ = g_s_ at a VPD of 0.79 kPa.

### Leaf water potential

Leaf water potential was measured using the Scholander Technique (Model M-1505D, MMM Tech Support, Berlin, Germany) during September/October 2019. The measurements were conducted using at least five leaves per tree species, each at predawn (4:30–5:30), noon (12:30–13:30), and pre-dusk (17:00–18:00).

### Stem circumference measurements

Circumferential change was measured with logging band dendrometers (LBDs) with a built-in thermometer (DRL26, EMS Brno). Dendrometers were attached at breast height on the stems and data were recorded every 30 minutes. In total, 29 Dendrometer were mounted at the study site.

### Sap flux measurements

The thermal heat dissipation method of Granier [51, 52] was used to monitor sap flux independently or concomitantly with leaf transpiration. Sap flux sensors (Heinz Kauper GmbH, Bayreuth, Germany) were installed at breast height (1.3 m) on the stems of up to three individuals of each studied species. Three pairs of sensors were equidistantly inserted 2.8 cm deep into the stem of the investigated trees. The protruding parts of the three sensors were shielded with aluminum foil to protect the probes from damage and external temperature fluctuations. From each pair of sensor probes, the upper one was heated by a constant power supply from a transmission line. The difference in temperature between the two probes (ΔT) was recorded continuously every minute and averaged over 10 minutes by a multiplexer connected with a data logger (AM16/32B and CR800, both Campbell Scientific Ltd., Logan, UT, USA).

The sap flux density J_s_ (g H_2_O cm^-2^ sapwood min^-1^) is related to ΔT by: J_s_ = 0.714 U^1.231^ [51] and U = (ΔT_0_–ΔT)/ ΔT. ΔT_0_ is the value of ΔT, when sap flux is considered zero. Zero sap flux was determined as the lowest nighttime value of ten diurnal sap flux cycles. For a rough comparison of the daily water consumption of the trees, sap flux density, accumulated over 24 h, was multiplied by the hydroactive sapwood area (J_s_ × sapwood area). Actively conducting sapwood areas of the studied trees were deduced from a sapwood-versus-DBH relation determined with five to eight conspecifics of different diameters on the research plot. A dye (0.2% aqueous solution of indigo carmin; Roth, Karlsruhe, Germany) was injected into the stems of these trees through holes drilled with a 5-mm stem borer (Suunto, Vaanta, Finland). The trees were injected at 1.3 m height in two positions in different directions. After 2 hours, core samples were collected from 2–3 cm above the dye injection points [53, 54]. The thickness of the core samples colored by the dye was used to calculate the active sapwood area using the equation of concentric circles (πR^2^-πr^2^), where R and r are the radii of the entire stem and the unstained inner part of the cross-section, respectively.

### Timing of measurements

Because of logistical constraints in the mountain rainforest (temporary power failure, breakdown of internet connection, defaults of equipment, etc.), not all measurements could be conducted during the same time periods (S1 Table). This concerns mainly the measurements of leaf gas exchange. However, these data show performances of leaves under the anyhow continuously changing weather conditions and are not specific for a particular season or year. Sap flux records accompanied all measurements and thus can be used to link the measurements performed at different times.

### Data analysis

Statistical analyses were performed using OriginPro 2019, R (4.0.5), and RStudio (v.1.3.959). All statistical tests were considered significant when P < 0.05. For sap flux determination the 10-day moving window function of TREX R package, version 1.0.0 by Peters et al. [55] was used to screen possible nocturnal sap flux. Dendrometer data were edited and analyzed using self-written scripts and DendRoAnalyst [56].

### Inclusivity in global research

The foundation “Naturaleza y Cultura Internacional (NCI)” permitted and assisted to build the towers in the Reserva Biológica San Francisco. The Ministerio de Ambiente of the Province Zamora Chinchipe allowed the research under Document No. 021-IC-FAU/FLO-DPZCCH-MA. Additional information regarding the ethical, cultural, and scientific considerations specific to inclusivity in global research is included in the S1 File.

## Results

### Ecological factors affecting the hydrology of the mountain rainforest

**Fig 3:**
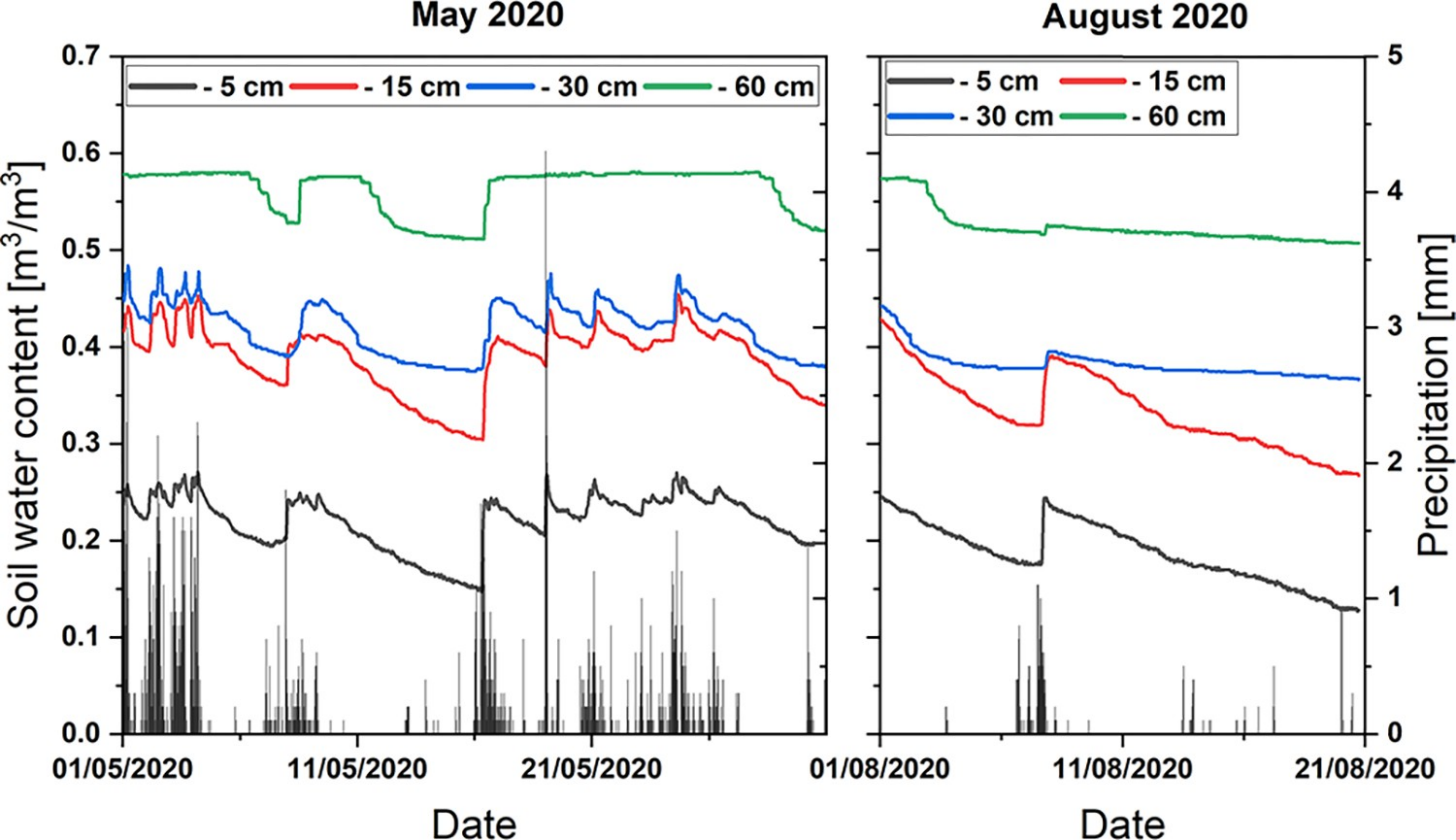
Soil water content at 1980 m a.s.l. in the research area at various soil depths over a wet (May 2020) and 3 weeks of an exceptionally dry month (August 2020). The vertical bars represent precipitation events of different intensities.

**Diurnal dynamics of soil water content.** SWC increased with soil depth from around 25% in the top mineral layer to 50–55% at 60 cm (Fig 3). In the uppermost 30 cm, it reflects the dynamics of the rainfall, while below 30 cm depth SWC amplitudes decreased. At 60 cm depth, SWC remained consistently above 50% even after an exceptionally long spell of days without rain as in August 2020.

#### Diurnal dynamics of air temperature and VPD

**Fig 4:**
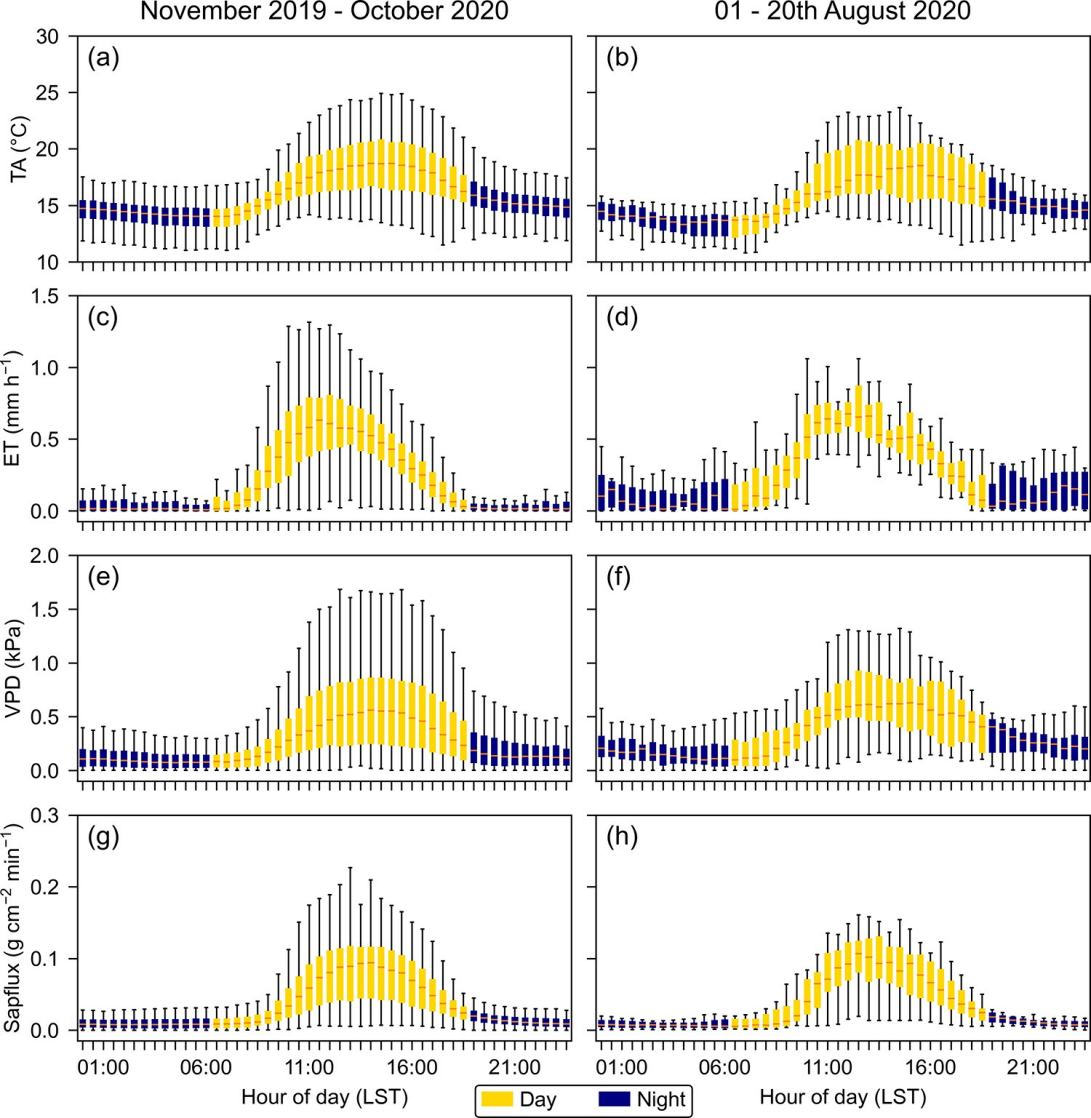
Diurnal courses of above-canopy air temperature (TA,°C), evapotranspiration (ET, mm h^-1^), vapor pressure deficit (VPD, kPa), and sap flux (g cm^-^^2^ min^-1^). The left column shows the time period November 2019 –October 2020, right column August 1–20, 2020.

Averages of daily courses of air temperature and VPD were calculated for a long-term 12-month period (November 2019 to October 2020) and an exceptionally dry month (August 2020), which received only one-third of the long-term mean (Fig 4A and 4B, see also Fig 3). Nighttime air temperatures ranged between 12 and 20°C, whereas during the day 25°C were frequently exceeded in sunny hours. Commonly, leaf temperatures adapted readily to the air temperature, except, when hit by sunflecks. VPD was generally low, rarely exceeding 1.5 kPa during the day and 0.5 kPa during the night (Fig 4E and 4F).

#### Diurnal dynamics of ET (Fig 4)

ET was the by far dominating hydrological flux, amounting to 79% of the precipitation of the research area. In contrast to air temperature (TA) and VPD, ET reached its maximum already before noon (Fig 4C). This phenomenon held also for the exceptionally dry August 2020 (Fig 4D). The considerable scatter of the climate variables results from the long observation period of up to 12 months, and indicates a high day-by-day variability not only of ET, but also of VPD and air temperature. Average ET was consistently positive, providing evidence that water vapor release into the atmosphere occurred not only during daytime (4.4 mm/day on average equivalent to 85% of total daily ET) but also during the night (0.8 mm, ~15%). During the dry August 2020, both daytime and nighttime ET increased by 0.8 and 0.6 mm, respectively, and their ratio shifted in favor of the latter (from 15 to 22%, respectively): More than one-fifth of the total daily ET occurred during the night.

#### Diurnal dynamics of sap flux and stem circumference changes

The diurnal course of leaf transpiration of canopy trees could be measured only for a limited number of species whose crowns were accessible via the towers. To examine a larger number of tree species over longer times, the diurnal dynamics of xylem sap flux was investigated. For comparison with ET (Fig 4C and 4D), the sap flux dynamics during 24 hours, averaged from 10 tree species over one year as well as during the extremely dry month of August 2020 are shown (Fig 4G and 4H). Overall, the daily dynamics of sap flux correlated better with VPD (r = 0.81) than with ET (r = 0.61). Sap flux dynamics showed a delay of around one hour against VPD (Fig 4E and 4F) and particularly against ET. However, when analyzed with a time-lagged cross-correlation of 60 minutes, it showed the highest correlation with ET (r = 0.74). The median (10 species, one year) of nocturnal sap flux decreased during the first half of the night, passed through a minimum around midnight, and increased slightly towards the morning. Like ET, also the rates of daytime sap flux were higher during the dry August 2020, but the ratio of day- to nighttime sap flux was identical for both (85:15%), the long-term and the August 2020 data.

**Fig 5:**
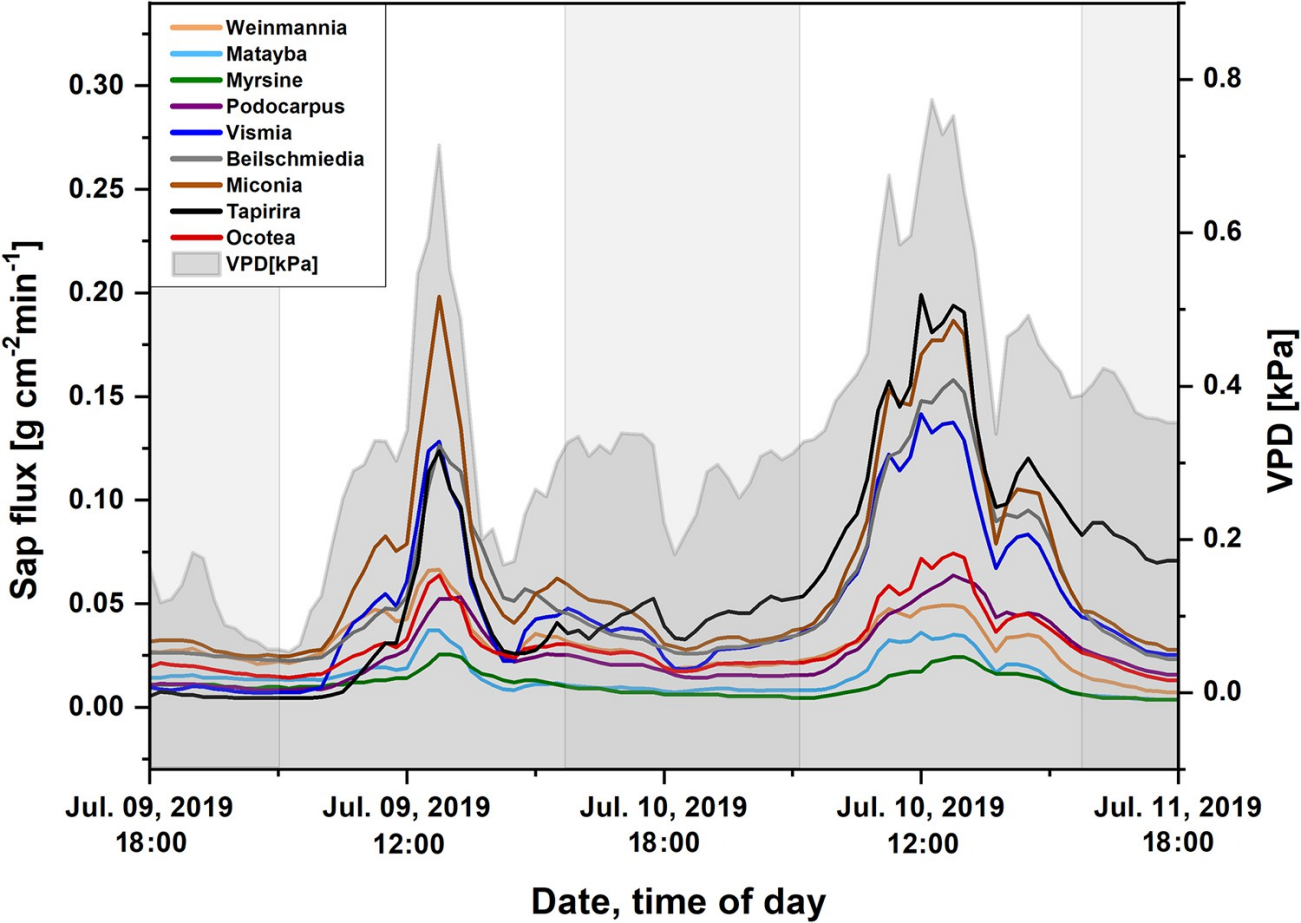
Normalized sap flux (g cm^-2^ min^-1^ at a depth of 2.8 cm) of individuals of nine tree species growing on the research plot in the evergreen mountain rainforest in the Reserva Biológica San Francisco. The data are means of the recordings of three sensors per tree. The grey background shows the atmospheric water vapor pressure deficit (VPD). Vertical light grey bars indicate night hours. (n = 9).

The potential contribution of individual tree species to the total stand ET was estimated from the daily sums of sap flux (Table 2). Species with high sap flux rates were *Tapirira guianensis*, *Vismia canavillesiana*, *Miconia calophylla*, and *Beilschmiedia tovarensis*, while low sap flux rates were recorded in *Weinmannia microphylla*, *Matayba inelegans*, *Myrsine coriacea* and *Ocotea aciphylla* (Fig 5).

**Table 2:** Distribution of the diurnal sap flux in eight evergreen tree species over day and night and average water potential of the leaves. n.m. = not measured. ± indicates standard errors.

Species	Total daily sap flux (gram x cm^-2^ x d^-1^)	Percent sap flux (%)			Mean leaf water potential ±SE (MPa)
Time of day		6:00–19:00*	19:30–0:00	0:30–6:00	
*Miconia calophylla*	16.13	77.4	12.3	10.2	-0.2 ±0.04
*Tapirira guianensis*	15.54	81.9	12.3	5.7	-0.3±0.03
*Vismia canavillesiana*	14.38	86.6	8.5	4.9	-0.4±0.02
*Beilschmiedia tovarensis*	13.82	79.2	12.1	8.7	n.m.
*Matayba inelegans*	4.82	71.9	12.9	15.2	-0.6±0.01
*Podocarpus oleifolius*	6.96	74.0	15.5	10.5	-1.0±0.07
*Weinmannia microphylla*	8.42	72.2	14.2	13.6	-1.2±0.04
*Ocotea aciphylla*	6.55	66.1	16.8	17.1	-1.4±0.03

*Transition periods (e.g., 19:00 to 19:30) were omitted.

**Fig 6:**
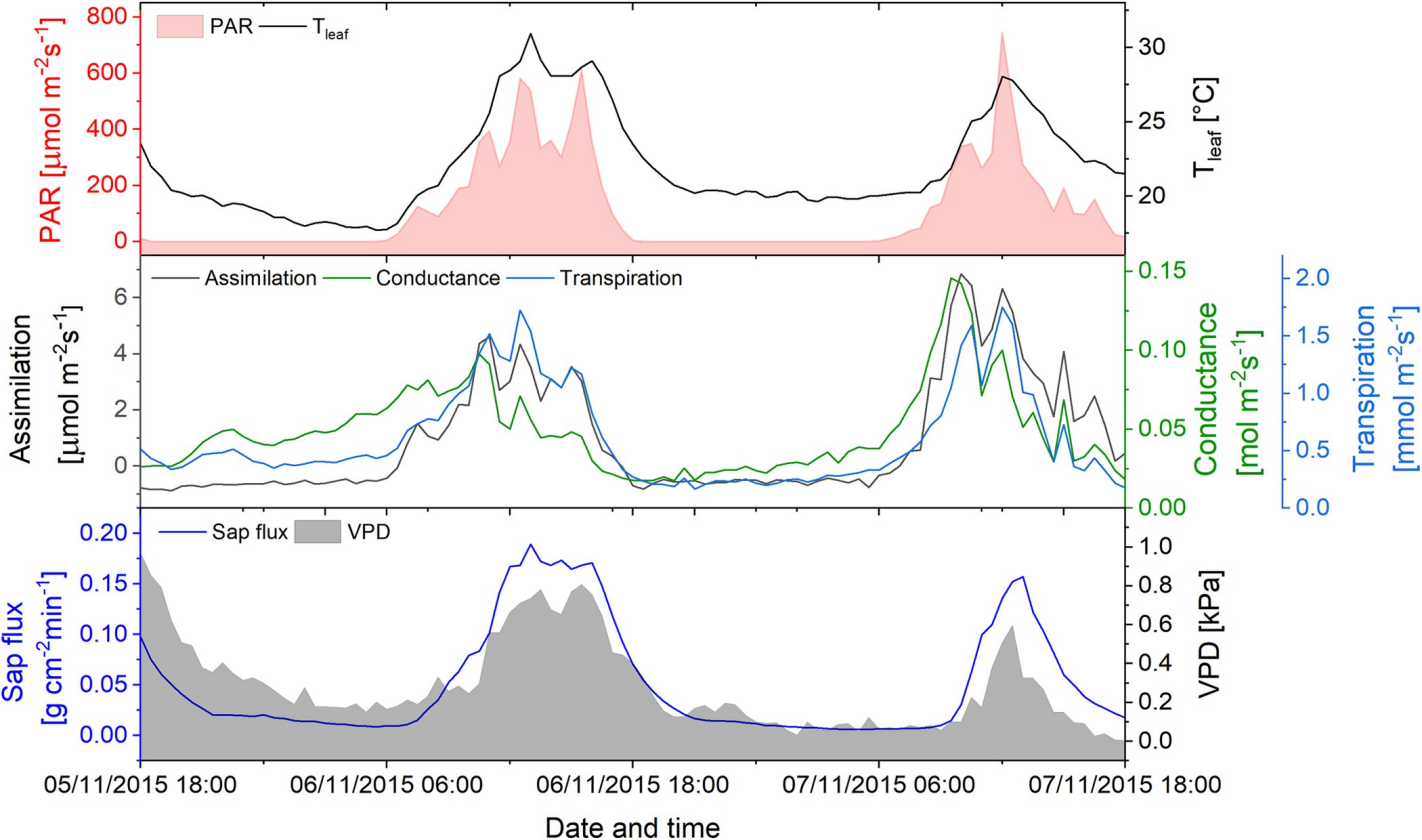
Gas exchange on the background of microclimate parameters (VPD–shadowed in grey, PAR–red area) of a leaf in the sun crown of *Tapirira guianensis*, measured over 2 days (November 5–7, 2015) under simulated natural atmospheric conditions with the IRGA. Simultaneously, sap flux was measured in the stem at breast height (lower panel).

The averaged sap flux decreased during the first half of the night to a minimum after midnight (Fig 4E and 4F). The subsequent increase towards the end of the night can be attributed to the tree species with high sap flux rates (*Miconia*, *Tapirira*, *Beilschmiedia*, and *Vismia*) and may indicate nocturnal transpiration (see Fig 6). The leaves of these tree species showed a relatively high (less negative) average water potential, while the leaves of the trees with low sap flux rates (*Ocotea*, *Podocarpus*, and *Weinmannia*) operated at a low (numerically more negative) leaf water potential. *Matayba* was in between both groups, showing a low daily sap flux concomitant with a medium average leaf water potential (Table 2).

**Fig 7:**
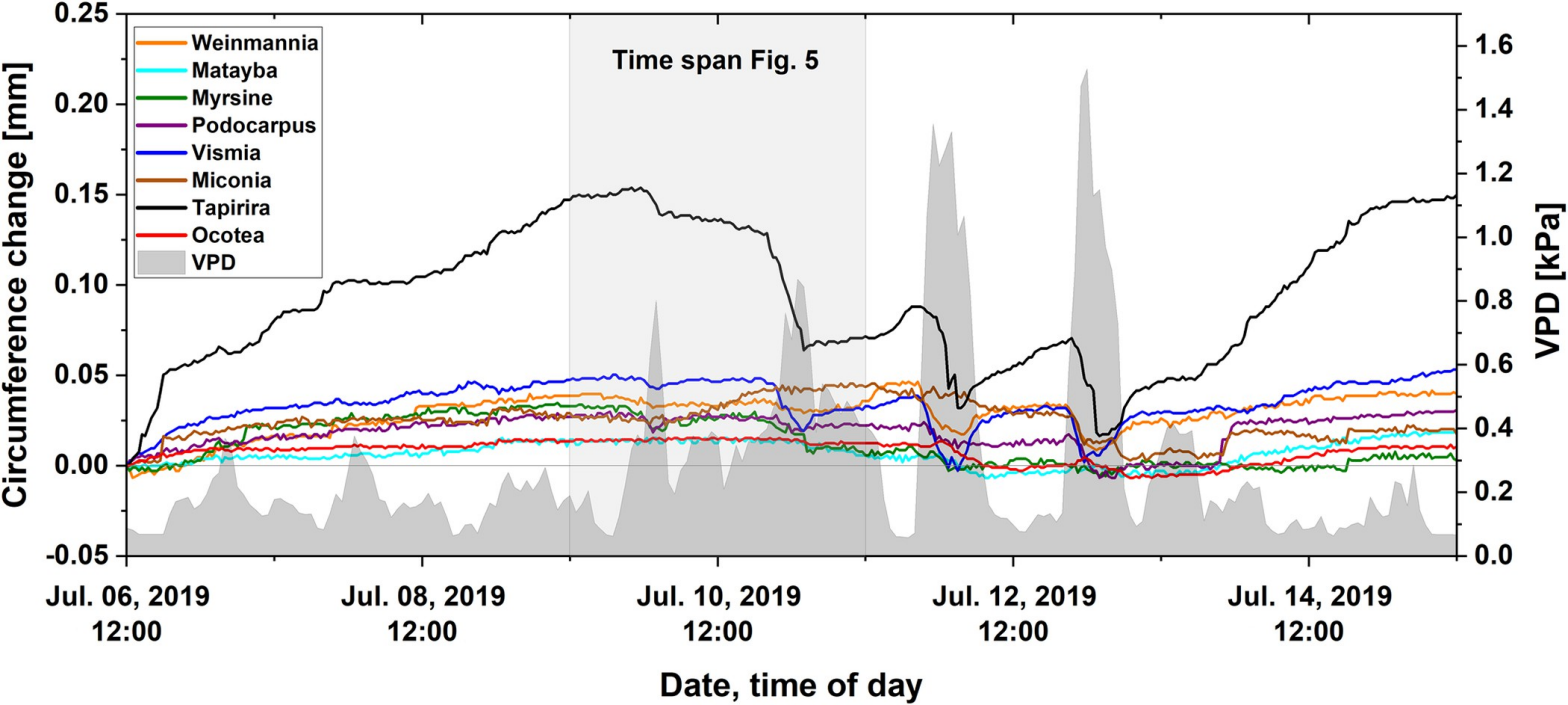
Stem circumference changes of different tree species during a short spell of dry weather. The grey background shows the atmospheric water vapor pressure deficit (VPD). The time span shown in Fig 5 is shaded (n = 29).

Shrinkage of the stem diameter was especially visible during dry periods. Water stress was released when the water supply exceeded the loss from transpiration, usually during late afternoon and early night hours. During the night, a clear replenishment effect was visible in all species. With the onset of precipitation, most species reached the pre-drought maximum after several days (Fig 7).

#### Diurnal course of gas exchange and hydrology of leaves

*Tapirira guianensis* is one of the big canopy species with a share in the species composition of the research plot of 6%. Together with *Miconia* it showed the highest rates of sap flux density of all investigated tree species and therefore was chosen for a detailed analysis of nocturnal transpiration. Fig 6 shows one of the longest uninterrupted gas exchange measurements of *Tapirira* leaves under simulated free-air conditions. The highest leaf conductance measured with *Tapirira* leaves *in situ* during daytime was 0.33 mol m^-2^s^-1^, and therefore the data recorded also from day 2 of the experiment (November 7, 2015, g = 0.15 mol m^-2^s^-1^) were meaningful. In spite of a relatively low VPD, the leaves of the investigated individuals (n = 2) showed continuous conductance for water vapour and considerable transpiration during the night. Nighttime transpiration contributed up to 20.7% to the (maximum) daily water consumption of 33.6 kg d^-1^ (Table 2). The courses of nocturnal conductance and transpiration were strongly positively correlated (r = 0.88) but opposite to the trend of VPD.

Daytime transpiration correlated nearly linearly with PAR (r = 0.89 Table 3), but less strongly with VPD (r = 0.57). Due to clouds, PAR was moderate during these days, not exceeding 750 μmol quanta m^-2^ s^-1^. Plotting daytime leaf conductance versus VPD showed several hystereses, thus precluding a meaningful correlation coefficient. During nighttime sap flux followed VPD until a minimum shortly before dawn (Fig 6). In contrast, the courses of SFn and transpiration did not correlate, whereas a positive correlation was observed during daytime hours (r = 0.59) with a time-shift of one up to 2.5 hours, depending on the weather conditions.

**Table 3:** Parameters characterizing leaf transpiration over two nights and two days of *Tapirira* and *Ocotea* as representatives of the two groups of differing hydrological performance. *p < 0.05.

Species	Total water consumption per day [kg]	Night			Day				
		Correlation coefficient		Percent	Correlation coefficient				Percent
		g/VPD	E/VPD	E_N_/E_tot_	g/VPD	g/PAR	E/VPD	E/PAR	E_D_/E_tot_
*Tapirira guianensis*	33.6	0.08	0.48*	20.7	-0.20	0.31*	0.57*	0.89*	79.3
*Ocotea aciphylla*	19.3	-0.11	0.00	7.5	-0.46*	0.44*	-0.13	0.73*	92.5

**Fig 8:**
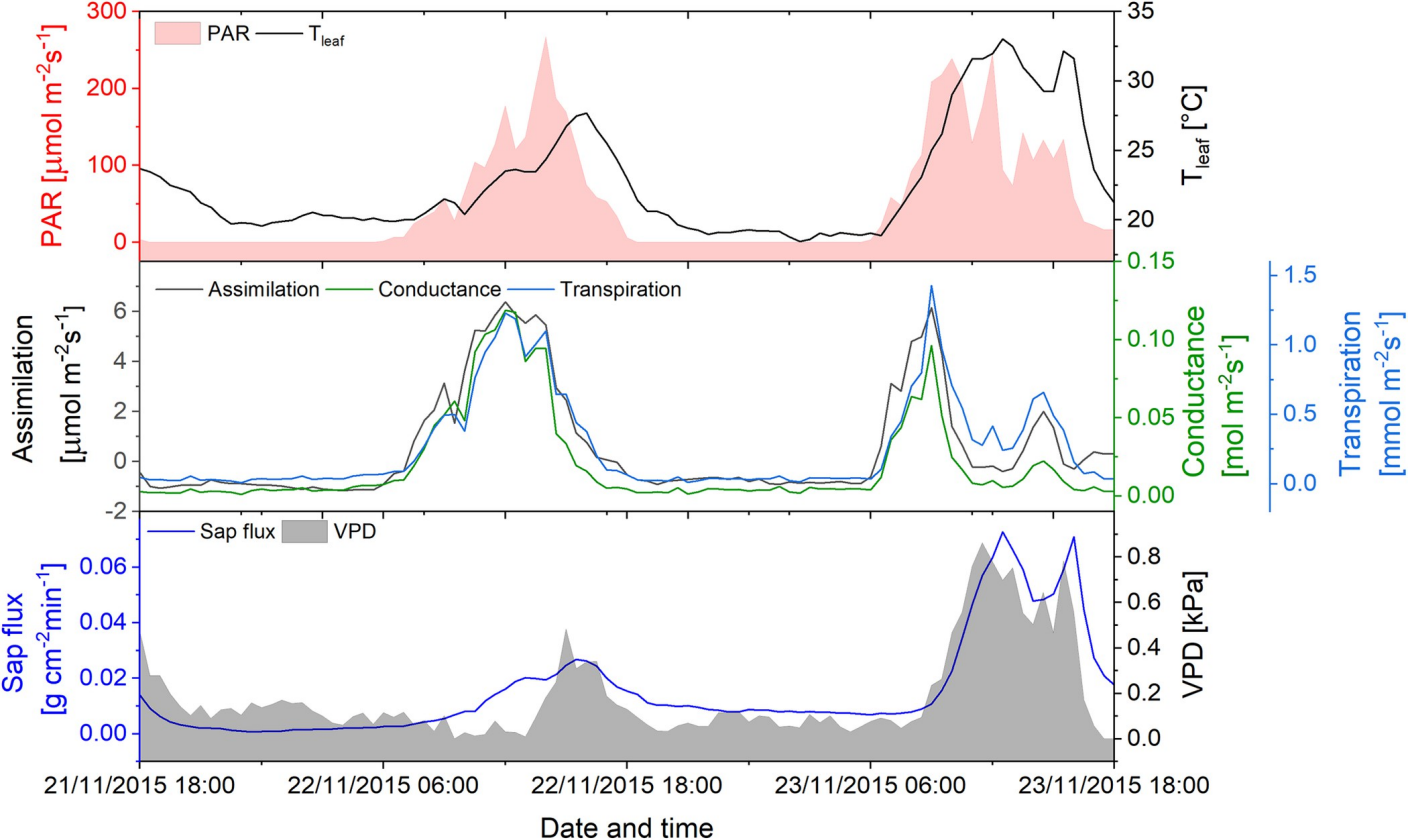
Leaf gas exchange on the background of microclimate parameters (VPD and PAR) of *Ocotea aciphylla*, measured over 2 days (November 21–23, 2015) under atmospheric conditions simulated as close to natural as possible with the IRGA.

*Ocotea aciphylla* is a representative of trees with low daily water consumption. The investigated individual is a canopy tree comparable to the examined *Tapirira* specimen. There are at least five species of *Ocotea* on the core plot with a total share of 4.8% of the tree individuals. Fig 8 shows leaf gas exchange for two consecutive days (November 22–23, 2015). Maximum daytime leaf conductance on the second day (0.17 mol m^-2^ s^-1^) was still close to the highest measured daily maximum (0.25 mol m^-2^ s^-1^), and therefore the data are considered meaningful. During both nights (19:00–6:00 h), rates of transpiration were near zero and NTT thus accounted for not more than 5.3% of the total diurnal transpiration (Fig 8). Averaged over 16 diurnal courses, it was 7.5%. Like in *Tapirira*, nighttime conductance was strongly correlated with NTT, but, in contrast to that species, not with VPD. Daytime transpiration rates of up to 1.3 mmol m^-2^ s^-1^ (on a dry morning temporarily up to 3.5 mmol m^-2^ s^-1^) were in the known range of sclerified leaves [57] and correlated well with PAR, but not with VPD due to hysteresis. Normalized sap flux rates correlated closely with VPD during the day, but negatively during the night (p<0.01).

The higher average rate of leaf transpiration (130 ±102 mmol m^-2^ d^-1^, n = 6) in *Tapirira* corresponded well with its higher average leaf conductance (g_average_ = 0.05 ± 0.007 mol m^-2^ s^-1^; n = 6 days). The corresponding values of *Ocotea* leaves were 110 ± 88 mmol m^-2^ d^-1^ (n = 14) for average transpiration and 0.03 ± 0.0013 mol m^-2^ s^-1^ (n = 15) for leaf conductance.

#### Factors controlling nocturnal transpiration (Table 3)

**Fig 9:**
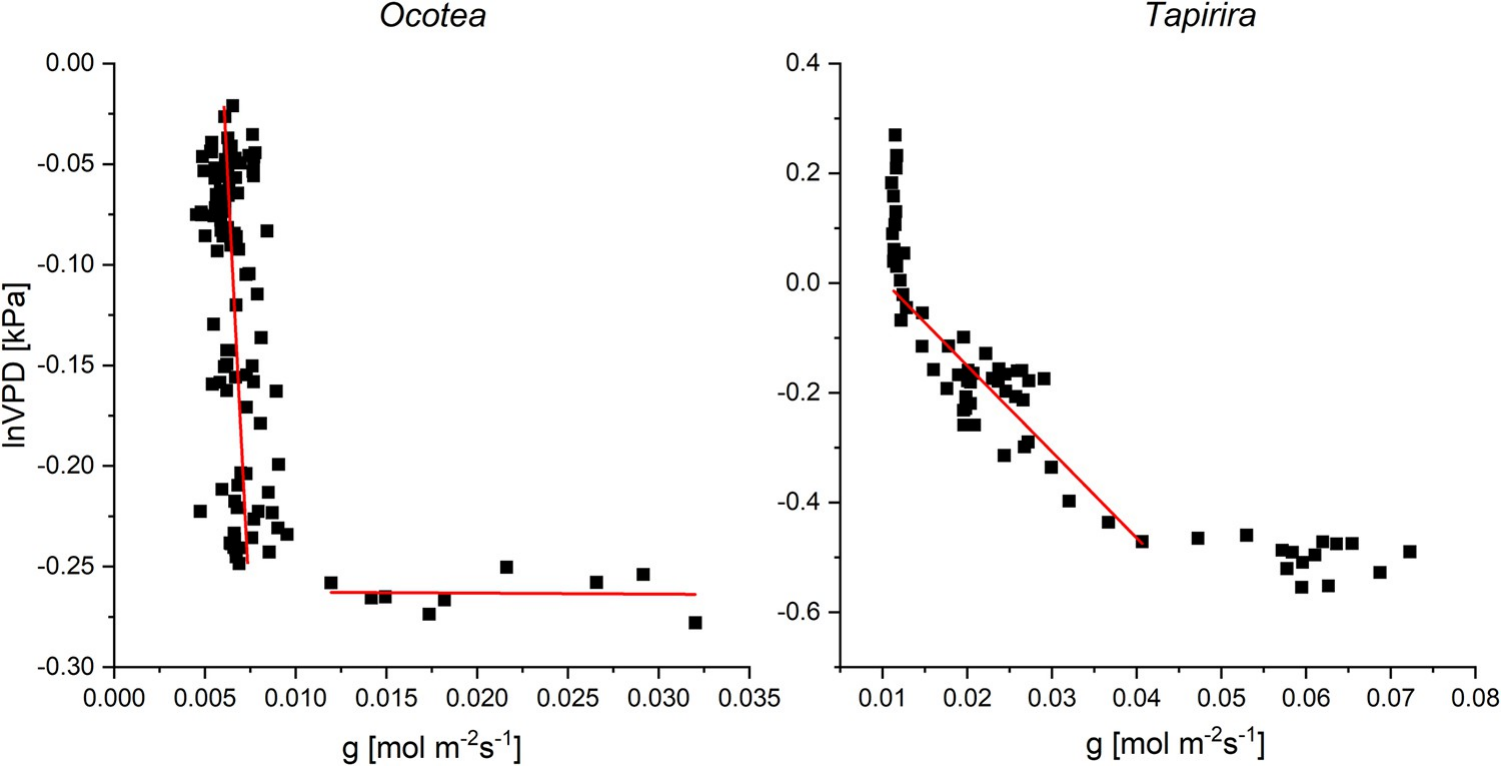
Nighttime (19:00 to 6:00) response of leaf conductance (g_leaf_) to lnVPD (following Oren et al. 1999) of *Tapirira* and *Ocotea*, respectively.

During the night, the stomata of *Tapirira* leaves were at least partly open, and transpiration responded positively to VPD in the range between 0.6 and 1.3 kPa. In that range, the sensitivity (m) of g_s_ to the atmospheric water vapor deficit was 0.055 mol m^-2^s^-1^ln(kPa)^-1^. At higher VPD, the stomata progressively closed. Calculating VPD for complete stomata closure by the function e^b/m^ (when b = g_sref_, Oren et al 1999) results in 1.27 kPa, which is close to the observed value (Fig 9). At VPD lower than 0.6 kPa g_s_ varied, but did no longer respond to changes of VPD. The leaves of *Ocotea* showed a particularly low leaf conductance (and transpiration) during the night and there was no clear dependence of either variable to VPD (Table 3). The minimum nocturnal conductance (~5 mmol m^-2^ s^-1^) was half that of *Tapirira* (Fig 9). There was an abrupt transition between the VPD range in which the stomata closed (VPD ≥ 0.8 kPa) and the range in which the stomata were slightly open but did not respond to VPD (≤ 0.8 kPa). The average sensitivity of nocturnal g_s_ to VPD, as calculated by equation (1), was only 0.0029 mmol m^-2^s^-1^ln(kPa)^-1^ and hence physiologically irrelevant, as was the value for complete closure of the stomata (9.7 kPa).

## Discussion

### The plot level: Diurnal dynamics of evapotranspiration

Due to the dense vegetation cover of the mountain rainforest, evapotranspiration is considered to result from plant transpiration (and interception shortly after a rain event). Rain showers and the incidence of fog transitorily suspended the ECov measurements, but under these conditions, the air was saturated with moisture and thus transpiration cannot be expected. Wind speed in the research area was generally low, especially during the night (1–1.5 m s^-1^), rarely exceeding 2 m s^-1^ even in the windiest month August [58], and thus possible effects of turbulences on the interpretation of the ECov measurements can be neglected. Given the low VPD and wind speeds near the surface, evaporation from the soil surface is assumed negligible. The share of 15% of total diurnal ET released from the forest during the night suggests ongoing transpiration by at least part of the vegetation. Compared to the globally estimated share of nocturnal transpiration between 6.3 and 7.9% [38], this value appears rather high; however, high rates of nocturnal transpiration between 25 and 80% have also been recorded at higher elevations in the Atlantic tropical rainforest [28].

Comparing the accumulated amount of daily evapotranspiration with the amount of rainfall over one year (Nov. 2019 –Nov. 2020) revealed that the bulk of the rain and fog water received by the forest is returned to the atmosphere via ET (78.8% of the annual precipitation of 2016 mm). Fleischbein et al. [59] estimated an ET of 62% for the same forest, composed equally of plant transpiration and interception. Relating measured and modeled surface runoff, respectively, to rainfall (without fog precipitation), Wilcke et al. [60] calculated ET in three catchments of the research area between 54 and 62% with an interception share of 70%. These and our results contrast with the result of a model calculation for the year 2000 [40], which stated that the main portion of rainwater leaves the ecosystem via run-off or subsurface flow. The model resulted in stand transpiration between 1 and2.4 mm per day, which is substantially less than the measured long-term ET mean reported in this study (5.15 mm). In the exceptionally dry period in August 2020, the overall balance between precipitation and ET was even negative: Over three weeks, total precipitation of 27.6 mm contrasted with an ET of 133.77 mm, resulting in an over decrease of the soil water content (Fig 3, right panel) by a simultaneously increased transpiration of the vegetation.

Following irradiance, the diurnal courses of air temperature and VPD reached their maxima in the early afternoon. In contrast, the daily maximum of ET was observed during the late morning hours, indicating that ET did not directly follow VPD. The discrepancy resulted from the early peaking of transpiration (Figs 6 and 8) and the subsequent midday depression, as a response to the increasing VPD (e.g. Fig 8, November 23). SWC of the upper 30 cm soil decreased between rainfall events (Fig 3). According to Moser et al. [61], the fine roots of the trees are concentrated in this layer and thus are subjected to the fluctuations of the water supply, which eventually can trigger signaling of water shortage to the leaves. During the prolonged drought in August 2020, some trees already wilted, in spite of their sclerophyllous leaves.

### The tree level: Water relations and hydrological types

Compared to trees in semi-arid tropical or temperate regions, the daily water consumption even of the bigger trees in the tropical mountain rainforest was low. In spite of an average LAI between 4 and 6 [62] for *Tapirira*, the tallest of the investigated trees, a water consumption of only 33.6 kg was estimated on a rainless day. *Ocotea*, as an example of the water-saving group of canopy trees, consumed only up to 19.3 kg of water per day. Motzer et al. [39], also commenting on the relatively low water consumption, reported a daily water throughput of 5.0 ± 2.5 kg for a smaller unidentified *Ocotea* tree in the research area (DBH 12.7, height 13 m). The low daily water throughput is understood as a response to the low VPD, proven by the long-term median below 1 MPa even during the sunny hours of the day (Fig 4). The tree species investigated in this study are mid-successional species, which follow an overall conservative water use strategy, in contrast to pioneer trees like *Cecropia montana*, which was reported to consume up to 120 kg H_2_O per day [39]. In the present study, stomatal conductance fluctuated over the daytime between 0.006 and 0.25 mol m^-2^ s^-1^ and thus was in the range known for tropical trees [39, 63, 64].

With respect to water relations, plants have been classified into iso- and anisohydric types [65]. Although the genetic background of such intrinsic properties has been questioned [66, 67], and a continuum between both behaviors has been proposed instead [68], the expanded hydricity concept of Meinzer et al. [69] has proven useful in plant ecohydrology. Anisohydric performance has been shown for part of the trees of a moist tropical forest in Borneo [70, 71], while isohydric behavior appears to prevail in an Amazon lowland forest [72]. Regarding the trees investigated in this study, isohydric-like performance could be attributed to *Ocotea* and anisohydric-like to *Tapirira*: It exhibited a comparatively high water consumption, and its leaves showed a relatively high predawn water potential (Table 2) with a considerable amplitude of diurnal fluctuation by 114%. In contrast, *Ocotea* leaves, operating at a low predawn water potential, exhibited a considerably smaller diurnal oscillation (50%), and the tree had a substantially lower daily water consumption. The other, above-mentioned hydrological traits of both species corroborate their behavior as anisohydric-like and isohydric-like, respectively. With respect to water consumption, leaf gas exchange, and the range of leaf water potential, *Vismia canavillesiana*, and *Miconia calophylla* behaved like *Tapirira*, whereas, *Matayba inelegans*, *Podocarpus oleifolius*, and *Weinmannia microphylla* concurred rather with *Ocotea aciphylla* (Fig 5, Table 2).

The hydrological behavior affected also the extent of the diurnal changes of the stem circumferences (Fig 7), although these may also partly be skewed by stem growth. *Tapirira*, *Vismia*, and *Weinmannia* showed transpiration-related stem contraction during daytime followed by relaxation during the subsequent night, while the trees in the *Ocotea* group hardly showed any diurnal changes in stem circumference.

### The leaf level: Conductance, transpiration, and xylem sap flux

During the daytime, leaf conductance and especially transpiration correlated best with light intensity (Table 3), due to the light stimulus on the stomata [73]. During nighttime, endogenous or other environmental factors control stomatal conductance [8]. In the VPD range between 0.6 and 1.1 kPa, *Tapirira* leaves responded to an increasing VPD with a decrease in leaf conductance (Fig 9) suggesting an effect of the water potential gradient between the leaf interior and the atmosphere on the regulation of the stomata also in the dark. The sensitivity of nocturnal leaf conductance to VPD was 55 mmol m^-2^ s^-1^ln(kPa)^-1^, which is in the range presented by Oren et al. [50] as an average (~60 mmol m^-2^ s^-1^ln(kPa)^-1^ for many species during daytime. During daytime, sensitivity was lower (48 mmol m^-2^ s^-1^ln(kPa)^-1^ due to the overriding light signal on the stomata [73]. Considering the results reported by Barbour & Buckley [74] for *Ricinus*, the lower day-time sensitivity is typical for well-watered high-light plants. The minimum leaf conductance of 11 mmol m^-2^ s^-1^ recorded at VPDs higher than 1.1 kPa was by a factor of 10 higher than an average cuticular permeability for evergreen broadleaf woody plants [75], suggesting some leakiness of the stomata [76]. Moreover, uncontrolled nocturnal water loss was facilitated by the high water potential of the leaves. At VPD below 0.6 kPa, nocturnal leaf conductance deviated from the near-linear relation between g and lnVPD. Non-linearity of that relation during nighttime has been reported for trees of the montane Atlantic rainforest of Brazil [39], however with a tendency to linearity towards lower VPDs, which is in contrast to our findings. Stressing the complexity of stomata regulation by environmental factors, the authors held back from presenting a physiological explanation. The same holds for this study which favors the gross idea of a VPD threshold at ~0.6 kPa, from which the VPD gradient across the leaf epidermis is the dominating factor in nocturnal stomata regulation. Overall, the time-course of leaf transpiration shows a positive correlation with that of VPD in the day as well as at night (Table 3). In the group of trees with anisohydric-like leaf performance *(Miconia*, *Tapirira*, *Beilschmiedia*, and *Vismia*), a significant share of SFn took place during the early night hours (Table 2) with decreasing flux rates until a minimum at midnight or even later, suggesting mainly replenishment of the water potential in the trees’ living tissues. The increase of the flux rates in the second half of the night in that group of trees might be due to an increasing NTT after the recovery of the leaves’ turgor (see Fig 5). Diverting at least part of SFn into NTT was related to the average water potential of the leaves: *Miconia*, *Tapirira*, and *Vismia* operated at a (numerically) high, *Ocotea*, *Podocarpus*, and *Weinmannia* at a more negative leaf water potential (Table 2).

As indicated by transpiration rates less than 2% of the day-time maximum, the stomata of the *Ocotea* group closed during the entire dark period even at very low VPDs. This can be concluded from the sudden transition between the VPD range in which the stomata closed (VPD ≥ 0.8 kPa), and the range in which the leaves showed very low conductance but did not respond to changes in VPD (≤ 0.8 kPa, Fig 9). Consequently, calculation of the sensitivity of nocturnal leaf conductance to VPD appears irrelevant. This notion is in line with the lack of a correlation between nocturnal leaf conductance and transpiration with VPD (Table 3). The observed minimum conductance of 4.5 mmol m^-2^ s^-1^ is but a little higher than the published value of 1 mmol m^-2^ s^-1^ for cuticular transpiration of evergreen broadleaf trees [75]. The more negative leaf water potential of these trees performing isohydric-like leaf hydrology might contribute to throttle uncontrolled water loss. During daytime, the sensitivity of leaf conductance to VPD was also low, but at least in the order of magnitude found in previous studies [50]. The trees with isohydric-like leaf hydrology exhibited very low rates of nocturnal sap flux. Due to the lower transpiration rates and the smaller diurnal change of the leaf water potential, recharging of the trees’ internal water reservoirs could have been achieved already by the end of the day.

In contrast to the different nocturnal performances, daytime leaf conductance and transpiration correlated well in *Tapirira* and *Ocotea* (R^2^ between 0.7 and 0.91, respectively). This is plausible since correlations of conductance and transpiration with light intensity (PAR) were stronger than with VPD (Table 3, see also [39]). In contrast, leaf conductance and transpiration correlated only weakly with sap flux during the daytime, due to a delay of the latter. On the assumption that the internal water reservoirs of the trees were replenished in the course of the night, the duration of the lag between leaf transpiration and sap flux could *inter alia* reflect the volume of that water reservoir, which initially feeds transpiration. Sap flux during daytime correlated better with VPD than with leaf transpiration. This phenomenon has been observed also in earlier studies [39, 77], but its physiological cause is still not well understood. As shown by Strobl et al. [78], the gas exchange of individual leaves in a crown shows different time kinetics depending on the position in the crown and varying light conditions. Xylem sap flux, measured beneath the first branches integrates over the water demand of the entire crown and thus is less depending on the regulation of the individual leaves’ transpiration.

## Conclusions

Overall mean annual evapotranspiration, directly measured over more than a year, accounted for 79 percent of the total precipitation and was thus the major hydraulic flux in the ecosystem. Its daily course proves the release of water vapor from the vegetation into the atmosphere also during the night and the increase of ET during periods of drought, mainly by enhanced nocturnal transpiration. Due to the closed vegetation cover of the forest, evapotranspiration must have resulted almost entirely from the transpiration of the trees.

Despite the high humidity in the mountain rainforest, anisohydric-like as well as isohydric-like performance of canopy trees were observed in the study plot of about 0.25 ha at 1900 m altitude. An important difference was found in the conductance of the leaves for water vapor during nighttime: Trees with anisohydric-like performance showed nocturnal transpiration sustained by a measurable conductance that responded at least partly to VPD, whereas isohydric-like species closed their stomata in the dark. Nighttime evapotranspiration of the forest was thus attributed to the share of the trees with anisohydric-like performance.

The coexistence of similar-sized canopy trees with anisohydric- and isohydric-like water relations suggests the idea of a physiological trait corresponding to the mosaic climax of the tropical mountain rainforest.

## Supporting information

S1 Table Overview of analyzed measurement and observation periods of different ecological and meteorological parameters and their temporal resolutions. (DOCX)

S1 File Inclusivity in global research. (DOCX)
