# Supplementary material for: Trees with anisohydric behavior as main drivers of nocturnal evapotranspiration in a tropical mountain rainforest
Source: PLoS One. 2023 Mar 31;18(3):e0282397. doi: 10.1371/journal.pone.0282397 (PMC10065286; doi:10.1371/journal.pone.0282397)
Supplement: S1 Table — (DOCX) [file pone.0282397.s001.docx]

Table S1 Overview of analysed measurement and observation periods of different ecological and meteorological parameters and their temporal resolutions

| **Parameter** | **Measurement period** | **Observation period** | **Temporal resolution** |
| --- | --- | --- | --- |
| Soil water content | September 2018 – October 2021 | September 2018 – October 2021 | 5 min |
| Eddy Covariance | November 2019 – October 2020 | November 2019 – October 2020 | 30 min |
| Sap flux | 2019, July 9 – July 10 | March 2016 –  November 2019 | 30 min |
| Stem circumference | 2019, July 6 – July 15 | March 2016 –  October 2020 | 30 min |
| Leaf water potential | 2019, September - October | 2019, September - October | Predawn,  midday & evening |
| Leaf gas exchange | 2015, November 5 – November 7  2015, November 21 – November 23 | Nov 2015 | 10 min |
